# Reducing childhood stunting in India: Insights from four subnational success cases

**DOI:** 10.1007/s12571-021-01252-x

**Published:** 2022-04-01

**Authors:** Rasmi Avula, Phuong Hong Nguyen, Lan Mai Tran, Supreet Kaur, Neena Bhatia, Rakesh Sarwal, Arjan de Wagt, Deepika Nayar Chaudhery, Purnima Menon

**Affiliations:** 1 International Food Policy Research Institute, South Asia Office, New Delhi, India; 2 Independent Researcher, Hanoi, Vietnam; 3 Previously With NITI Aayog, Government of India, New Delhi, India; 4 Government of India, NITI Aayog, New Delhi, India; 5 UNICEF, New Delhi, India; 6 World Bank, New Delhi, India

**Keywords:** Stunting, Multisectoral actions, Nutrition interventions, Household conditions

## Abstract

Global success case analyses have identified factors supporting reductions in stunting across countries; less is known about successes at the subnational levels. We studied four states in India, assessing contributors to reductions in stunting between 2006 and 2016. Using public datasets, literature review, policy analyses and stakeholder interviews, we interpreted changes in the context of policies, programs and enabling environment. Primary contributors to stunting reduction were improvements in coverage of health and nutrition interventions (ranged between 11 to 23% among different states), household conditions (22–47%), and maternal factors (15–30%). Political and bureaucratic leadership engaged civil society and development partners facilitated change. Policy and program actions to address the multidimensional determinants of stunting reduction occur in sectors addressing poverty, food security, education, health services and nutrition programs. Therefore, for stunting reduction, focus should be on implementing multisectoral actions with equity, quality, and intensity with assured convergence on the same geographies and households.

**Supplementary Information:**

The online version contains supplementary material available at 10.1007/s12571-021-01252-x.

## Introduction

Globally, the past decade has witnessed an unprecedented focus on undernutrition, particularly on stunting, which gained traction with the extension of ambitious World Health Assembly's (WHA) 2012 nutrition target of 40% reduction in the number of children under 5 who are stunted (World Health Organization, 2014) by its inclusion into the Sustainable Development Goals (United Nations, 2015). This in turn created demand for guidance on achieving stunting reduction at the national and subnational levels.

There is an extensive body of literature examining various determinants of stunting. Analyses based on large-scale data sets have found that stunting is associated with maternal stature (Li et al., 2020; Wali et al., 2020) and education (Dorsey et al., 2018; Li et al., 2020; Subramanyam et al., 2010), household wealth (Li et al., 2020; Subramanyam et al., 2010) and sanitation status (Larsen et al., 2017) across multiple countries and in India. Recent evidence from various geographies indicates changes in a combination of these determinants contributed to improvements in height-for-age Z scores (HAZ). For example, improvements to maternal and newborn health had contributed to 28% change in HAZ in Senegal (Brar et al., 2020) and in Peru (Huicho et al., 2020) but only 11.5% in Nepal (Conway et al., 2020). Improved maternal nutrition contributed to 26% change in HAZ in Peru (Huicho et al., 2020), but only 5% in Ethiopia (Tasic et al., 2020), and parental education contributed to 25% change in HAZ in Nepal (Conway et al., 2020), 20% in Peru (Huicho et al., 2020), and only 15% in Senegal (Brar et al., 2020). Improvements to economy contributed to 19.5% changes in HAZ in Senegal (Brar et al., 2020), but only 9% in Nepal (Conway et al., 2020) and 4% in Ethiopia (Tasic et al., 2020). Findings from these national level case studies highlight the importance of parallel improvements in maternal factors, socioeconomic conditions, coverage of health and nutrition interventions, under broader political and enabling societal conditions for achieving declines in stunting. These findings also indicate that there could be no one or same solution for addressing stunting. There are, however, few examples of similar case studies at sub-national levels (Haddad et al., 2014; Kim et al., 2017; Kohli et al., 2017b, 2020), despite broad recognition of intra-regional variations within countries in social and economic development (Sinha, 2003), and in nutrition (Kumar et al., 2020).

India, a nation contributing to a third of global burden of undernutrition, saw a decline in stunting between 2006 and 2016. Improvements in maternal nutrition and education, children’s diet, assets, open defecation, age at marriage, antenatal care contributed the most to the differences in stunting prevalence between low and high burden districts (Menon et al., 2018). At the same time, inter-state variation explained 56% of the variation in district stunting prevalence, which is indicative of differences across states in administrative and governance approaches (Menon et al., 2018). Underlying political and institutional differences among the states arising from varied political landscape and resulting governance models affect state’s capacity for growth, programmatic responsiveness, and accountability, which in turn could affect the proximal determinants of nutrition outcomes (Harriss & Kohli, 2009). This heterogeneity in outcomes and various determinants across specific states needs further unpacking to identify elements of success that could be transferred to other states. Understanding the key conditions for success could provide insights into creating or stimulating conditions in other states and in similar low-and middle-income countries. Thus, our study contributes to learning about *how* to reduce stunting, and how policy and programs help improve determinants of stunting, particularly in a sub-national context. Our findings are applicable to other large countries, which may have national program and policy frameworks that play out in different ways in subnational units.

We sought to identify determinants of decline in stunting across select states by examining four questions: 1) How did determinants of child stunting change over time? 2) How did changes in these determinants contribute to decline in stunting? 3) What was done in terms of policies related to the key determinants that contributed to stunting decline and 4) what drove the policy (and other) changes?

## Methods

We identified three states– Chhattisgarh, Gujarat, and Odisha, which had the highest absolute percentage point (pp) decline in stunting, ranging between 11 and 15 pp between 2006 and 2016, to further our understanding of state-specific characteristics contributing to this positive change. We also selected Tamil Nadu as a historical success case for improvements in maternal and child nutrition.

Chhattisgarh is a newly formed state in eastern India with a population of ~ 30 million, with a third of its population below the poverty line. Gujarat is a state in western India with a population of 60 million and is one of the country’s high-income states. Odisha, a state in eastern India with ~ 47 million people, is one of the poorest states, and yet has been identified as a positive deviant in nutrition (Mohmand, 2012) and health related policies and programming (Thomas et al., 2015). Tamil Nadu, a coastal state on the deep south of India with 70 million population, is among the wealthier states in the country, with better infrastructure, governance, and social sector compared to several other states. It has consistently been a model state for development. For example, in 1998–99, stunting among children below three years in Tamil Nadu was 35% when the national average was 51%, and only 37.8% women married before 18 years of age in the state compared to 61% in the country.

We used mixed methods for the study. First, we used descriptive analyses to examine changes in stunting and its known determinants, then we conducted regression-decomposition analysis to examine the contribution of various determinants to changes in stunting between 2006 and 2016. Second, we constructed a timeline of evolution of program and policies associated with major drivers of change during the study period. Third, we interviewed stakeholders in the study states to understand their perceptions about the potential reasons for changes in key programs and policies. Finally, we integrated insights from all these research methods to identify the determinants and drivers of decline in stunting and examined similarities and differences across the four states. All analyses were conducted separately for each state.

### Examining changes in stunting reduction and its known determinants

We used two rounds of state level representative data from the National Family Health Survey 2005–06 (NFHS-3) and 2015–16 (NFHS-4) to examine changes in stunting and its various determinants for four states separately. These two data are representative at both national and state levels. Stunting was defined as height-for-age below two standard deviations of the WHO growth reference (HAZ < -2) (WHO, 2006). We conducted data quality assessment on HAZ and stunting measures (Supplemental Fig. 1). Using the UNICEF/Lancet conceptual framework (Black et al., 2013), we selected a set of immediate and underlying determinants, and interventions. The immediate determinants included maternal underweight (body-mass index BMI < 18.5), infant and young child feeding practices (early initiation of breastfeeding, exclusive breastfeeding, timely introduction of complementary foods, and adequate diet) and child disease (diarrhea in the last 2 weeks). The underlying determinants included maternal education, age at marriage, household social economic status (SES), religion, caste, sanitation, and village level sanitation and electrification. The household SES was constructed by applying principal component method to fifteen household assets to generate a factor score. We included coverage indicators for interventions during pregnancy (at least 4 antenatal care, consumption of 100 + iron-folic acid supplements, weighing, tetanus vaccination), at birth (skilled birth attendance), and during early childhood (full immunization, pediatric IFA supplementation, vitamin A, supplementation, and deworming). We compared changes in stunting and determinants between 2006 and 2016 using regression models, adjusting for the survey sampling design and applying sampling weights. All analyses used data for children under 5 years.

### Examining factors contributing to stunting reduction

To examine factors contributing to reduction in stunting, we first conducted bivariate analysis to examine association of each variable with stunting, then multivariable analyses after including immediate, underlying determinants, and nutrition‐specific interventions in the model. Third, we performed regression-decomposition analyses to assess how much of the change across determinants contributed to change in stunting. This analysis combines the analysis of differences in means of the explanatory variables (X) at the state level between 2006 and 2016 and regression estimates associated with these variables (βX) from a pooled regression model at the national level. We assumed that the direction of association between various determinants and stunting is similar across the states. Since stunting prevalence among children was within 0.2 to 0.8, we used linear regression rather than logistic regression (Cox & Snell, 1989; Hellevik, 2009). We also conducted sensitivity analyses, using HAZ (continuous variable) as an outcome. Based on the results of the decomposition analysis, we identified focus areas for policy analysis.

### Examining policy and programmatic changes

We conducted a literature review to analyze policy changes over the study period and to support overall analysis and interpretation. We reviewed published and grey literature, government documents, and websites to construct a timeline of program and policy implementation for each state between 2000 and 2016 (Kohli et al., 2020). We used specific search terms defined in Supplemental Table 1 and conducted the literature review between May and July 2021.

We also conducted semi-structured interviews with key stakeholders from the government, academia, civil society and development partners (n = 24 for Tamil Nadu and n = 17 for each of the other states) who were knowledgeable of the state context, its nutrition-relevant policies, and programs (Supplemental Table 2). Interviewees from the government included those who were in key positions when changes in stunting were observed. The interview guides were developed based on the policy timeline and were revised based on initial reflections from stakeholders (Supplemental Box 1). Interviews were recorded after obtaining consent from the respondents and were completed by December 2018. Confidentiality was ensured prior to beginning any interview. The transcripts were coded in *Excel* using a code list based on the interview guide, allowing for emergent codes. The codes were then clustered into broad thematic areas, and then summarized into select elements of the drivers of programmatic changes.

## Results

### Changes in child stunting and its known determinants

**Fig. 1:**
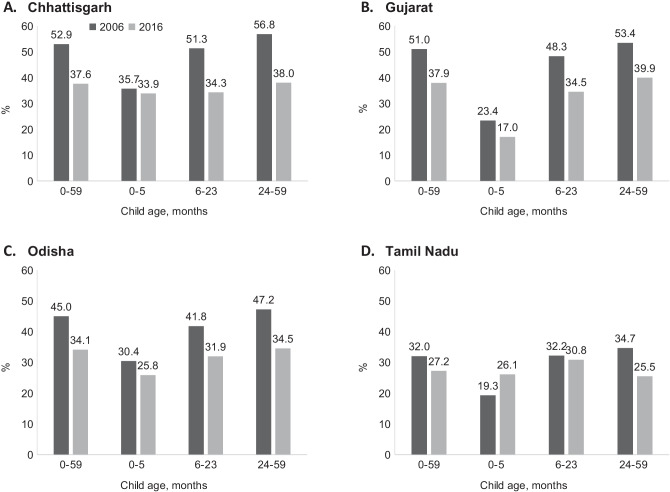
Changes in stunting between 2006 and 2016 in four states in India

Chhattisgarh, Gujarat, and Odisha had similar levels of stunting in 2006 (53%, 51% and 45%, respectively) (Fig. 1). Between 2006 and 2016, stunting declined in all three states (15 pp, 13 pp and 11 pp, respectively). In 2016, stunting ranged between 38% in Chhattisgarh and Gujarat to 34% in Odisha. There were only marginal declines in stunting among children < 6 months compared to children 6 to 23 months and 24 to 59 months between the two periods, therefore, we focused our analysis of contributors of change on children of 6–59 months of age in these states.

In contrast, stunting was lower in Tamil Nadu in 2006 (32%) compared to the three states and declined to 27% by 2016. The stunting reduction was mainly observed only among children 24 to 59 months (9 pp), but not among children < 24 months. Therefore, for Tamil Nadu, the analysis on contributors to changes was focused on children 24–59 months.

**Fig. 2:**
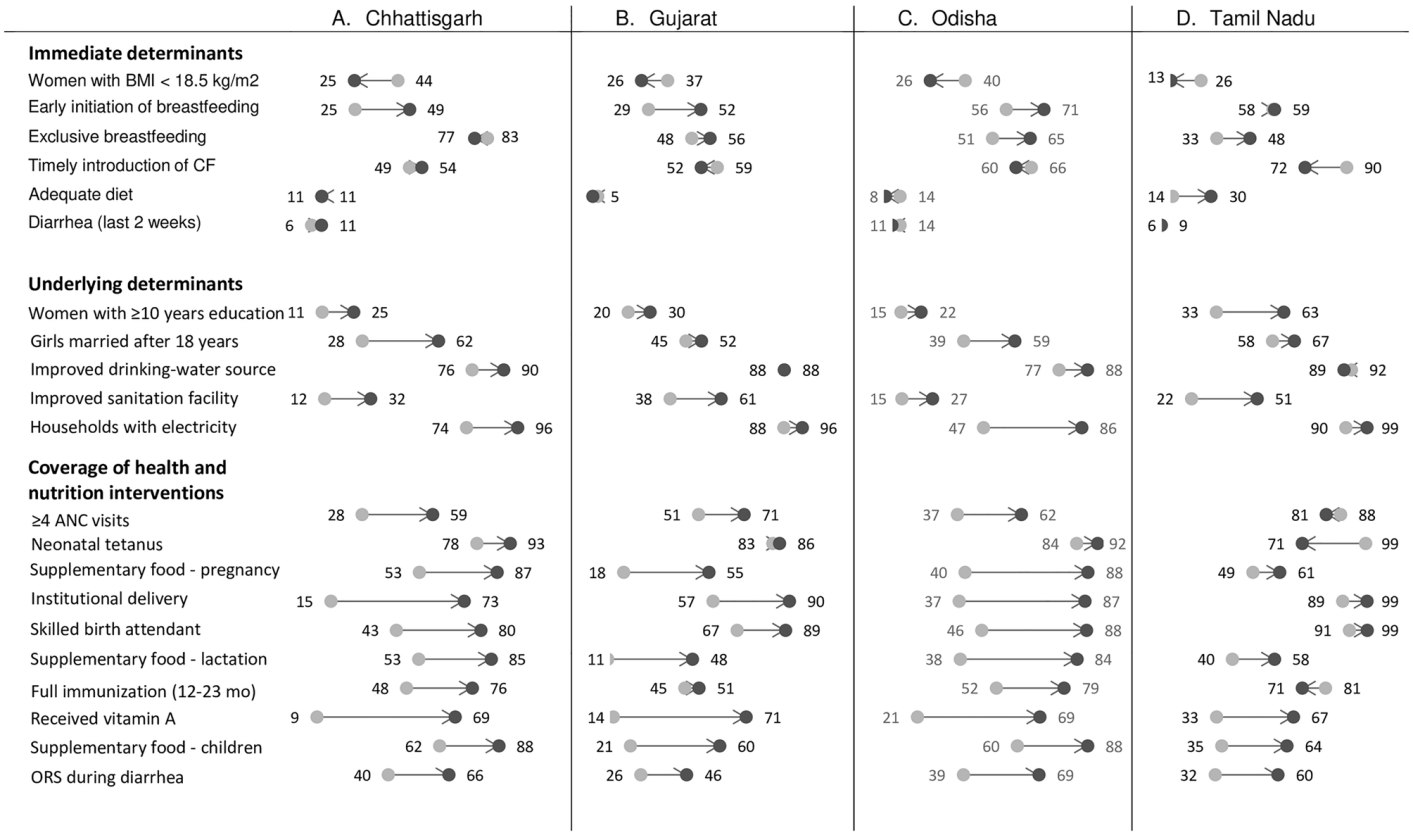
Multiple determinants improved in four states in India, between 2006 and 2016

Between 2006 and 2016, several known determinants of stunting improved in all states (Fig. 2). Among the immediate determinants, the proportion of women with low BMI declined in all four states, with change in coverage ranging between 12 percentage points (pp) in Gujarat to 21 pp in Chhattisgarh. Early initiation of breastfeeding improved over time in Chhattisgarh, Gujarat, and Odisha but not in Tamil Nadu. Adequate diet remained low and unchanged, except in Tamil Nadu (from 14 to 30%). Among the underlying determinants, access to sanitation facilities and electricity increased substantially in all three states. Chhattisgarh (32%) and Odisha (27%) had, however, a lower proportion of households using improved sanitation facilities. More girls were getting married after attaining the age of 18 years in all States. Although there was an improvement in women’s education, less than a third of women received 10 or more years of education in Chhattisgarh, Gujarat, and Odisha in 2016.

The coverage improved substantially for most health and nutrition interventions across Chhattisgarh, Gujarat, and Odisha. There was, however, interstate variability in the coverage of 4 or more antenatal care visits (ranging from 20 to 31 pp), protection against neonatal tetanus (3 to 15 pp), births attended by a skilled birth attendant (33 to 58 pp), children fully immunized (6 to 29 pp), and vitamin A supplementation (48 to 60 pp) (Fig. 2).

In Tamil Nadu, the prevalence levels across multiple determinants in 2006 were higher compared to other states and continued to improve. Maternal factors including women with low BMI declined (26% to 13%), while those with 10 or more years of education improved (33% to 63%). Households with access to sanitation increased (22% to 51%). Although, the coverage of some health and nutrition interventions improved, there were declines in antenatal visits (88% to 81%), protection against neonatal tetanus (99% to 71%), and full immunization (81% to 71%) in Tamil Nadu.

#### Contributors to stunting decline between 2006 and 2016

**Fig. 3:**
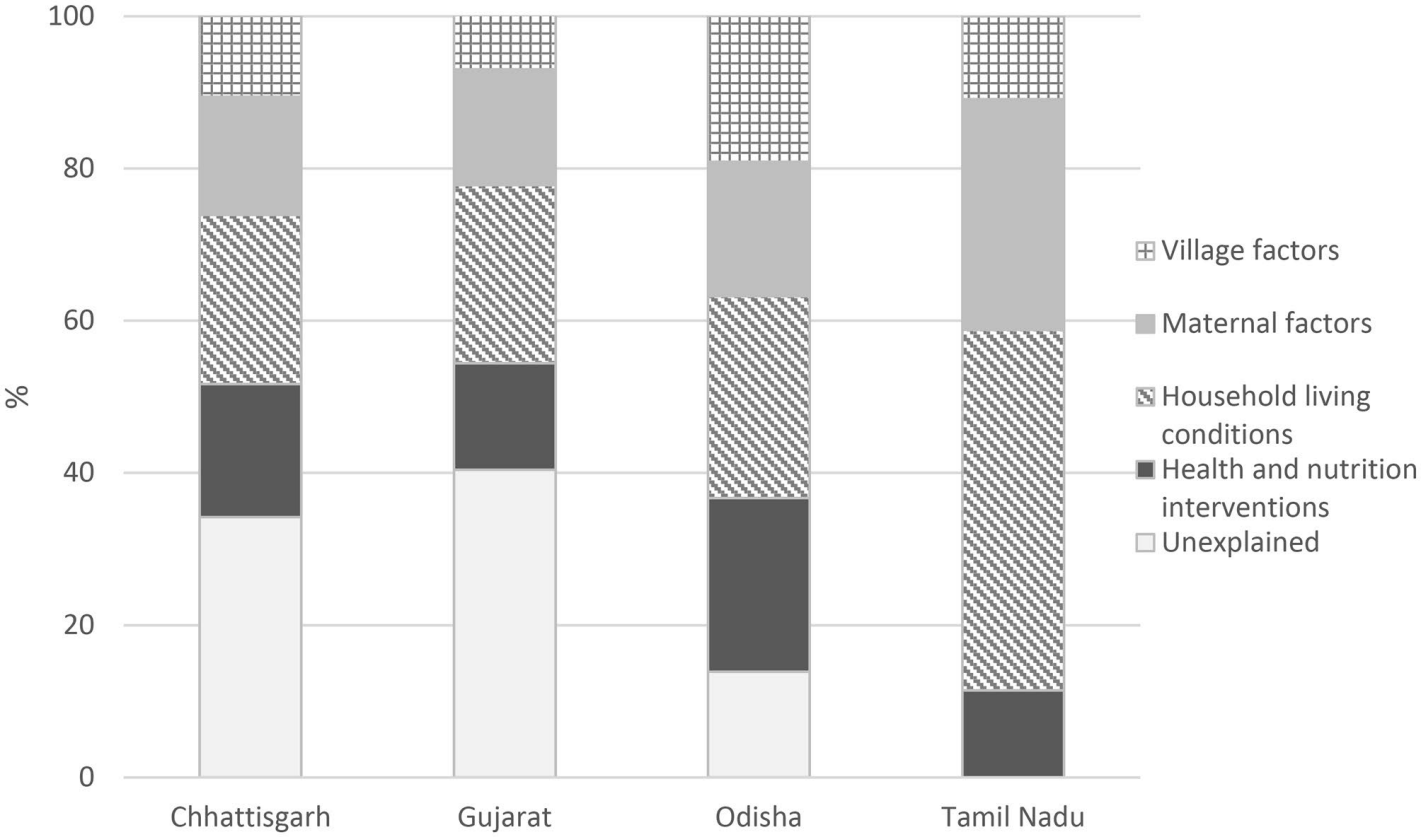
Multiple determinants across sectors contributed to changes in stunting between 2006 and 2016 in India

Across the four states, reduction in stunting between 2006 and 2016 was partially explained (66% in Chhattisgarh, 60% in Gujarat, 86% in Odisha and 100% in Tamil Nadu) by the variables in the model. These included improvements in the coverage of health and nutrition interventions (11% in Tamil Nadu, 14% in Gujarat, 17% in Chhattisgarh, 23% in Odisha), improvements in household-living conditions (22% in Chhattisgarh, 23% in Gujarat, 26% in Odisha, 47% in Tamil Nadu), and improvements in maternal factors (15% in Chhattisgarh and Gujarat, 17% in Odisha, 30% in Tamil Nadu). In addition, improvements at the village level factors contributed to between 7 to 19% of the stunting reduction across the four states (Fig. 3). The contribution of improvements in health and nutrition interventions was large in Odisha and Chhattisgarh, while changes to household conditions and maternal factors contributed the most in Tamil Nadu.

Sensitivity analysis using HAZ as an outcome showed similar findings (Supplemental Table 3). Overall, these analyses reaffirm that actions across multiple sectors are essential for achieving a decline in stunting.

## Policy and programmatic efforts targeting key determinants of stunting and their enablers

### Advancements in health and nutrition services

Our analysis of programmatic efforts indicates improvements observed in the coverage of health and nutrition interventions between 2006 and 2016 could be attributed to gradual programmatic evolution at the state level under an enabling national program mandate (Table 1). Between 2001 and 2007, India’s flagship program – Integrated Child Development Services (ICDS) was expanding with the aim to achieve universal coverage to provide basic nutrition and child development services. The National Rural Health Mission (NRHM), now referred to as NHM, was launched in 2005 to provide health services to rural poor with a focus on maternal and child health. These national programmatic expansions provided resources to bolster infrastructure and human resources within states.

**Table 1:** Evolution of policies and program within states under common national program framework

Programs	CHHATTISGARH	GUJARAT	ODISHA	TAMIL NADU
Health and nutrition programs	**2002: **Establish State Health Resource Centre (SOCHARA, 2005) **2002: **Mitanin (a friend) – volunteer health worker program (SOCHARA, 2005) **2012: **Caregiver managed daycare centers (Garg, 2014)	**2001/2002: **ICDS shifted to the Department of Women and Child development **2005: **Public–private partnership model to strengthen service delivery **2007: **Synchronization of departmental boundaries to ensure convergence of services	**2003–2004: **Odisha Health Vision 2010 **2004: **Positive deviance approach **2008: **Fee transportation for pregnant women **2009: **Adopted modified version of national fixed-day guidelines **2010: **Chief Minister’s nutrition council set up to facilitate convergence(Mohmand, 2012) **2011: **Mamata Abhiyaan: Maternity benefits program **2011: **Decentralization of food supplementation	**1980–1997: **Tamil Nadu Integrated Nutrition Project **1982: **Non-meal program **1995: **Pulse polio campaign – Polio free in 2004 **2003: **Malnutrition-free Tamil Nadu –Multi-sectoral strategy **2005: **Tamil Nadu health systems development project to reach marginalized and tribal population **2006: **Dr. Muthulakshmi Reddy Maternity Benefit Scheme **2008: **Comprehensive Emergency Obstetric and Newborn Care
Economy	•Poverty reduction slower than other states (World Bank, 2016a) •40% population below poverty line in 2012 •Rapid job growth in construction and service sectors (2005–2012)	•Significant progress in poverty reduction after 2005 •Improvements in forestry and logging, manufacturing, electricity, gas and water supply, transport, storage, trade and hotels •17% population below poverty line in 2012	•Poverty declined sharply after 2005 and was the fastest decline in the country •33% population below poverty line in 2012 •Poverty reduction faster than in other low-income states	•Fastest growing economy after 2005 •Per capita income doubled between 2004 and 2013 •Improvements in service sector •17% population below poverty line in 2012
Food security	**2001: **Allowed private participation for distributing subsidized commodities **2002: **Direct procurement of rice from farmers **2004: **De-privatization of private fair price shops to curb operational irregularities **2007: **Reduced the food grain price to below the central issue price(Krishnamurthy et al., 2014) **2007: **Chief Minister’s Food Assistance Scheme to increase the number of people entitled to the subsidized food program beyond the national program mandate **2012: **Portable smart card system to facilitate access to subsidized grains from any shop within a city **2012: **First state in India to pass the Food Security Act (Tillin et al., 2016)	•Implemented national PDS program	**2003**: Decentralized procurement to ensure price benefits to farmers **2008: **Subsidized rice at two rupees INR per kilogram **2008: **Supply chain computerized **2013: **Reduced the rice price to one rupee INR per kilogram	•Universal PDS
Sanitation	•Implemented campaigns to raise awareness about sanitation •Community approach to total sanitation	•**2005: **Implemented Total Sanitation Campaign •**2006: **Sanitation given prominence on state platform with a live broadcast of messaging by the state leadership to all villages in the state •State set a goal to cover all households by 2010 •Implemented awareness generations campaigns •Political leadership at the state level for sanitation	•Implemented national programs	•**1999–2004: **Implemented Total Sanitation Campaign in all districts •**2012: **Set a goal to eliminate open defecation by 2015 •**2012: **Envisioned coverage of all towns by underground sewerage system by 2017
Women’s education	•**2003: **National Programme for Education of Girls at the Elementary level (distinct component of universal education program) •**2004: **Kasturba Gandhi Balika Vidyalaya (KGBV); reach out to girls in marginalized social group, enrolment in grade 5 •**2004: **Saraswati Bicycle Supply Scheme •Construction of toilets for girls	•**2002– 2005: **Girls enrolling in first grade receive a bond for 1000 INR, and then receive bond plus interest upon completing 7^th^ grade •**2005: **Community awareness rally and campaign for girls’ education) in villages with less than 35% female literacy rate to increase enrolment •Residential schools for holistic development and life skill development of girls from •disadvantaged communities •**2005–06: **Life insurance program for each primary school student (85 lakhs), initiated after loss of children's lives in the 2001 Gujarat earthquake •Construction of School Sanitary Complexes •**2004–2005: **Computer education scheme in primary education, computer •**2012–13: **Supplementary nutrition provided to all primary school children •**2013–14: **Special training programme for out of school children	•Multiple initiatives until 2016 for education including self-defense program for girls, establishment of high schools	•**1997–2002: **Followed a 'package approach' to improve women's education (providing a package of concessions in the form of free supply of books, uniform, boarding and lodging, clothing for hostilities, mid-day meals, scholarships, free by-cycles) •**2011: **Marriage Assistance Scheme
Women-centric programs		•**2004: **Gender resource center	•**2001: **Self-help group program- Mission Shakti •**2011: **Reservation for women in local governance (Panchayati Raj Institution) increased from 33 to 55%	•**1992: **Cradle baby scheme •**1997: **Women’s groups

ICDS: Integrated Child Development Services, PDS: Public distribution system, INR: Indian Rupee

In Gujarat and Tamil Nadu, the policy focus was on improving maternal and child health and nutrition, whereas investments in nutrition and health programs in Chhattisgarh and Odisha were driven by focus on infant mortality. These four states implemented state specific initiatives between 2000 and 2016 to strengthen the reach and use of services (Table 1). Across the states, there was political stability, which facilitated continuity of programs. For example, in Odisha, one political party remained in power for three consecutive terms and bureaucrats were assured tenure to allow them to learn and implement the programs, facilitating implementation (Kohli et al., 2020; Menon et al., 2016). In Tamil Nadu, existence of the directorate of public health and infrastructure for program delivery provided additional support for effective planning of public health and its execution. In addition, development partners and civil society members played a critical role in providing technical and funding support for program implementation in these four states.

### Improvements in household conditions

Across the four states, it is likely that a combination of improvements in SES, food security, and sanitation created enabling conditions at the household level that supported reduction in stunting. Chhattisgarh and Odisha fall in the category of low-income states and Gujarat and Tamil Nadu rank among the high-income states; between 2005 and 2012, poverty declined, and economy improved in all these states, although at varied pace (World Bank, 2016b, 2017a, 2017b). The states differed in sectoral contributions to the economic growth, in job growth and female labor force participation. In Chhattisgarh and Odisha, farming continued to be the major employment source. In addition, national social-safety net programs such as the employment guarantee and the food subsidy programs likely contributed to improvements in households’ SES. Although, the national food subsidy program changed from being a universal to a targeted program, Tamil Nadu continued universal coverage (Kalaiyarasan, 2014), and Chhattisgarh (Kohli et al., 2020) and Odisha (Kohli et al., 2017a) undertook several reforms to effectively implement and expand the coverage of this program beyond the the national mandate.

All four states implemented national sanitation program. Chhattisgarh and Gujarat complemented the efforts with state-level initiatives to raise awareness and build community ownership. Gujarat’s state leadership gave prominence to the sanitation program. Tamil Nadu envisaged implementation of underground sewerage system and elimination of open defecation. Overall, in these three states, state-specific initiatives were undertaken (Table 1), which could have bolstered implementation of national programs and improved access to sanitation services.

### Improvements in maternal factors

Our empirical findings suggest improvements in maternal factors pertaining to age at marriage, maternal education, maternal BMI contributed to a reduction in stunting. Several national programs were initiated to increase girls’ enrolment in schools. Chhattisgarh, Gujarat, and Odisha also implemented several initiatives to improve girls’ education, including cash and kind incentives, special living accommodations for girls from the marginalized communities, grant of bicycles for commuting, and improving sanitation facilities for girls at schools (Table 1). Among the four states, Tamil Nadu had always had an inclusive and strong gender-focused developmental agenda, which resulted in multiple state initiatives to improve sex ratio at birth, girls’ education, raising age at marriage, improving women’s health and empowerment. In addition, three states also invested in improving women’s empowerment. It is likely that a combination of national programs, state initiatives to improve education and women’s empowerment facilitated improvements in overall maternal factors. Large effects were observed in Tamil Nadu, a state with the oldest legacy for investments in women’s care.

Several shared themes emerged across the states pertaining to the policy and programmatic efforts. Although there was no explicit focus on stunting reduction, states’ vision and priorities for improvements in its determinants drove investments in programs. This was partly facilitated by an enabling environment created by existing national level programs. The states’ high-level political leadership’s interest in the programs, bureaucratic capabilities, investments in state-specific initiatives, technical and financial support from development partners and civil society possibly contributed to the enabling conditions for improvements in the multiple determinants of stunting.

## Discussion

Between 2006 and 2016, considerable progress was seen in reduction of stunting among children aged 6–59 months in Chhattisgarh, Gujarat and Odisha, and among children 24–59 months in Tamil Nadu. The primary contributors to stunting reductions in these states were improvements in coverage of health and nutrition interventions (11–23%), household conditions (22–47%), and maternal factors (15–30%). A policy and program analysis highlights the critical importance of political and bureaucratic leadership, engaged civil society and development partners who together could have facilitated change in the programs and policies that targeted the key determinants.

Our findings, across states, reinforce the importance of delivering interventions in different sectors to tackle the multiple determinants of stunting. The need for multisectoral interventions, across programs and policies in the same geographies has been recognized before (Garrett et al., 2014) and our study lends additional empirical credence to this need.

Although the magnitude of contribution of various factors to stunting reduction varied across the states, improvements in household conditions contributed the most. These results are consistent with studies conducted in India (Nie et al., 2019), Bangladesh (Nisbett et al., 2017), and Nepal (Headey & Hoddinott, 2015). The next major contributor to decline in stunting was changes to maternal factors in Tamil Nadu and Gujarat, while improvements in the coverage of health and nutrition interventions contributed the most in Chhattisgarh and Odisha. Similar variability in factors contributing to changes in stunting were observed in other settings (Headey et al., 2017; Heidkamp et al., 2021). This variability across states and countries indicates the need for contextualized policy and programmatic initiatives to help focus the efforts in the sectors that need the most attention for continued decline in stunting. Our findings, together with those from other countries (Heidkamp et al., 2021) highlight that core sectoral actions to address known determinants of poor child growth are important drivers of change.

A common feature across the four study states was implementation of state-specific programs and innovations complementing national level efforts across health and nutrition programs, food security, sanitation programs, and women’s education. The possibility for evolving as exemplar states under a common national framework could be due to a combination of central programs, state-level choices and institutional innovations as demonstrated in the variation in industrial development across multiple regions (Sinha, 2003). India’s federal system of governance defines health, education, and sanitation as state subjects (Dash, 2007). Thus, states take the lead to address the varying burdens of malnutrition, while the national government remains responsible for setting policies, regulating systems, and funding national priority programs. There is variability in the focus among states on the programs implemented. For example, sanitation program was prioritized in Gujarat, whereas food subsidy program was prioritized by the leadership at the highest level in Chhattisgarh and Tamil Nadu.

Beyond the emphasis on programs, differences in state institutional mechanisms such as effective fund release and utilization (Choudhury & Mohanty, 2019) could have implications for effective program implementation among subnational regions. Similar gaps in implementation fidelity in decentralized health systems was observed in other settings (Eboreime et al., 2017). Further research is needed to examine the sub-national level agency and abilities to utilize national inputs effectively while adapting to local programming needs.

Our study underscores the need for continued focus on improving coverage and quality of the essential nutrition and health interventions, besides continuing to build linkages with social protection, livelihoods, agriculture and poverty reduction programs. Similar findings were noted across 11 countries, highlighting the multisectoral nature of undernutrition and the need to identify multisectoral actions (Heidkamp et al., 2021). We highlight the importance of high-level leadership for bringing focus to implementing programs targeting key determinants of stunting. This is aligned with the notion of commitment and capacity at various political and bureaucratic levels in decentralized settings as necessary ingredients for actions for improving nutrition (Gillespie et al., 2013).

Use of credible and high-quality multidimensional data on coverage, quality, scale, and nutrition outcomes enhances our understanding of nutrition problems and provides an empirical basis for evidence-based policymaking on nutrition.. In India, NFHS data and administrative data are used to track progress in nutrition and its determinants and to identify opportunities for improving program implementation (NITI Aayog, 2020). However, there are huge data gaps pertaining to food security, food consumption, and employment, which are important determinants of nutritional status. The periodicity of national level surveys varies, and it could be rendered non-informative for continued local-level programmatic decisions. These data gaps, however, could be addressed by using existing administrative data effectively for local level situation analysis and to develop strategies (International Food Policy Research Institute, 2014). Using further disaggregated data such as at the village level could enable targeting precise policy actions at the local level (R. Kim et al., 2021; Kumar et al., 2020).

Our mixed methods approach gives confidence in our findings and conclusions. The use of mixed methods provided state-specific insights on stunting reduction in the context of changes policies, programs and enabling environment in the last decade. While the decomposition regression analyses examined how the changes in determinants contributed to stunting reduction, the stakeholder interviews and policy analyses provided valuable information for supporting and complementing the regression findings. All these independent data sources were used to corroborate key messages and allowed for data triangulation on specific findings.

A few methodological notes are worth mentioning for data interpretation. The quantitative analyses used secondary national and state representative data, but such data lacks information on household food security, child infectious disease or mothers’ exposure to counselling. In these four-state case studies, we have not examined sub-state variability in nutrition outcomes, in program implementation, and the factors driving those differences. Further research is needed to deepen our understanding of sub-state level variability and to identify targeted solutions.

## Conclusions

To address malnutrition, it is imperative to ensure effective implementation of core, health and nutritional services while also ensuring poverty reduction, improving education, sanitation, and convergent delivery in geographies. Our study indicates the importance of policies, programs, state level priorities and the driving force of governance in triggering actions for improving determinants of nutrition. This highlights the need for systemic changes, which are long-term and should be initiated early, while continuing to work on effective program implementation, which could be improved immediately. For India and other similar LMICs to reach the WHA target for stunting, health and nutrition must continue to remain a development priority, particularly in the context of the COVID-19 pandemic and the rising of inequities. National, sub-national, and civil-society actors must continue working together to ensure sustained leadership for nutrition security and uninterrupted coverage, advocate for adequate financing, and emphasize on a collective multisectoral approach (Coalition for Food & Nutrition Security in India, 2020). Further research should focus on *how* to ensure implementation of existing programs with equity, quality, and intensity, and assured convergence on the same geographies and households.

## Supplementary Information

Below is the link to the electronic supplementary material.

Supplementary file1 (DOCX 465 KB)

## Data Availability

Data available upon request. We used the National Family Health Survey data, which are publicly available.
