# Supplementary material for: Reducing childhood stunting in India: Insights from four subnational success cases
Source: Food Secur. 2022 Apr 1;14(4):1085–97. doi: 10.1007/s12571-021-01252-x (PMC8975447; doi:10.1007/s12571-021-01252-x)
Supplement: Supplementary file 1 — Supplementary file1 (DOCX 465 KB) [file 12571_2021_1252_MOESM1_ESM.docx]

**Supplemental figure 1: Data quality assessment on height-for-age and stunting measurement**

| 1. **Chhattisgarh** |  |
| --- | --- |
| 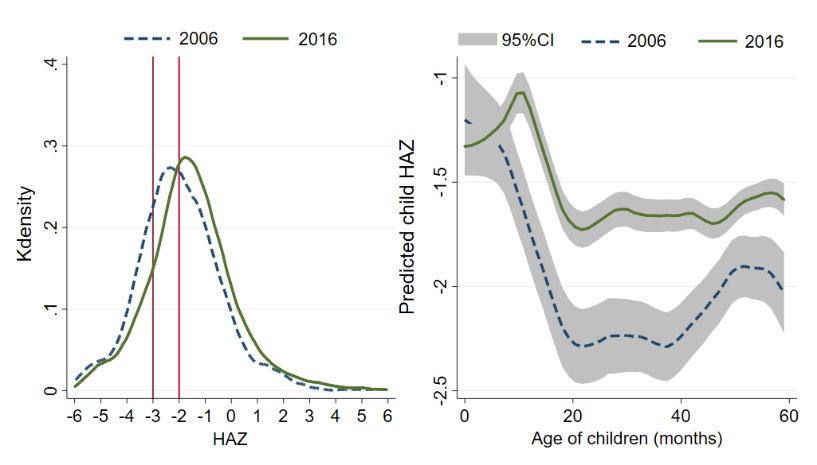 | \|  \| **2006** \| **2016** \| \| --- \| --- \| --- \| \| N \| 1592 \| 9283 \| \| Completeness (%) \|  \|  \| \| Age \| 91.3 \| 93.5 \| \| Height \| 89.9 \| 92.1 \| \| Weight \| 90.1 \| 92.2 \| \| Correct height measure position (%) \| 87.3 \| 98.5 \| \| Missing values (%) \| 11.4 \| 9.1 \| \| Stunting (%) \| 52.9 \| 37.6 \| \| HAZ (mean) \| -2.01 \| -1.52 \| \| HAZ (SD) \| 1.57 \| 1.58 \| \| Skewness \| 0.43 \| 0.49 \| \| Kurtosis \| 4.03 \| 4.26 \| |
| 1. **Gujarat** |  |
| 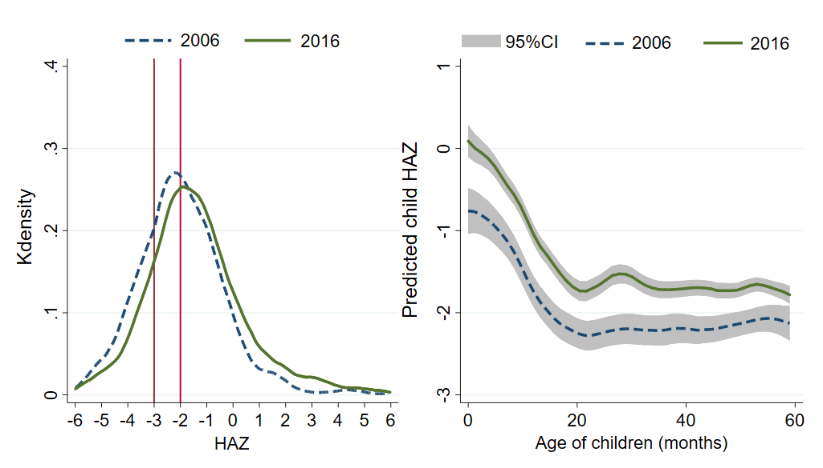 | \|  \| **2006** \| **2016** \| \| --- \| --- \| --- \| \| N \| 1571 \| 7730 \| \| Completeness (%) \|  \|  \| \| Age \| 93.3 \| 94.9 \| \| Height \| 89.6 \| 90.1 \| \| Weight \| 89.8 \| 90.5 \| \| Correct height measure position (%) \| 96.6 \| 94.8 \| \| Missing values (%) \| 12.3 \| 11.6 \| \| Stunting (%) \| 51.0 \| 37.9 \| \| HAZ (mean) \| -1.95 \| -1.35 \| \| HAZ (SD) \| 1.63 \| 1.83 \| \| Skewness \| 0.65 \| 0.73 \| \| Kurtosis \| 4.65 \| 4.22 \| |
| 1. **Odisha** |  |
| 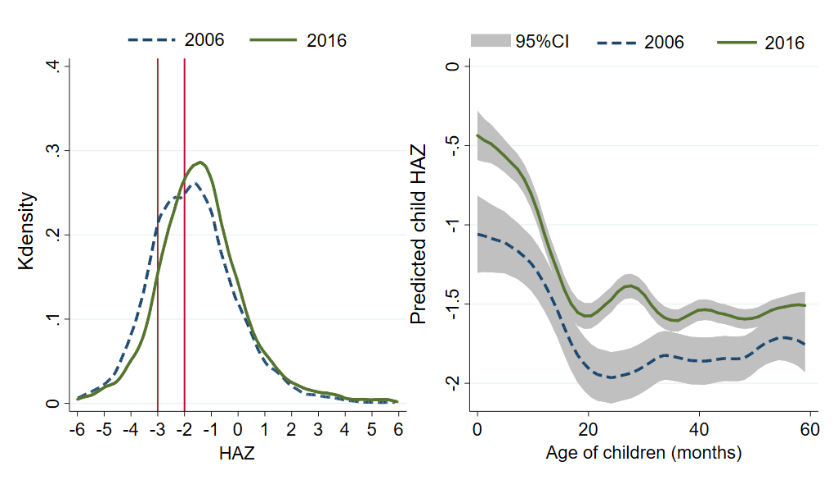 | \|  \| **2006** \| **2016** \| \| --- \| --- \| --- \| \| N \| 1781 \| 11106 \| \| Completeness (%) \|  \|  \| \| Age \| 92.2 \| 94.9 \| \| Height \| 88.8 \| 91.9 \| \| Weight \| 89.2 \| 92.1 \| \| Correct height measure position (%) \| 84.0 \| 96.2 \| \| Missing values (%) \| 12.8 \| 9.9 \| \| Stunting (%) \| 45.0 \| 34.1 \| \| HAZ (mean) \| -1.73 \| -1.31*** \| \| HAZ (SD) \| 1.58 \| 1.63 \| \| Skewness \| 0.52 \| 0.67 \| \| Kurtosis \| 4.02 \| 4.67 \| |
| 1. **Tamil Nadu** |  |
| 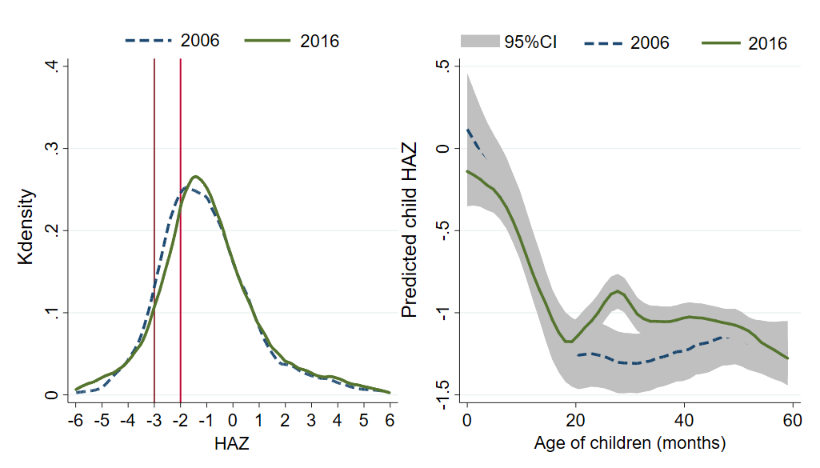 | \|  \| **2006** \| **2016** \| \| --- \| --- \| --- \| \| N \| 1735 \| 7922 \| \| Completeness (%) \|  \|  \| \| Age \| 95.6 \| 95.4 \| \| Height \| 90.3 \| 93.7 \| \| Weight \| 90.6 \| 93.8 \| \| Correct height measure position (%) \| 89.6 \| 94.6 \| \| Missing values (%) \| 12.6 \| 9.2 \| \| Stunting (%) \| 32.0 \| 27.2 \| \| HAZ (mean) \| -1.02 \| -0.92 \| \| HAZ (SD) \| 1.83 \| 1.87 \| \| Skewness \| 0.75 \| 0.58 \| \| Kurtosis \| 4.13 \| 3.96 \| |

Anthropometric measurement in large-scale surveys is a complex and difficult undertaking and inaccuracies in the quality of the anthropometric data arise frequently. This can have important implications for understanding the burden of malnutrition. In this paper, we examine data quality by 1) checking for the completeness of measurement for age, height, weight, 2) calculate the proportion of correct position for height measurement (lying down for children <2y and standing up for children ≥2y) and missing values, and 3) Dispersion of z-scores for height-for-age (HAZ) and test for normality. We found that overall data quality is reasonably good. In all states included in our study, the changes over time were real and occurred for means, distributions and for stunting prevalence.

**Supplemental Table 1:** **Search terms used for literature review**

| **Programs** | **Search terms** |
| --- | --- |
| ICDS | “ICDS”; “ICDS” AND “India”; “ICDS” AND “Odisha”/”Orissa*”; “ICDS” AND “Odisha”/”Orissa” and “nutrition” |
| NRHM | “NRHM”; “NRHM” AND “India”; “NRHM” AND “Odisha”/”Orissa”; “NRHM” AND “Odisha”/”Orissa” AND nutrition |
| PDS | “Public Distribution System”; “Public Distribution System” AND “India”; “Publish Distribution System” AND “Odisha”/”Orissa”; “Public Distribution System” AND “Odisha”/”Orissa” AND “nutrition” |
| NREGA | “MNREGA”; “NREGA” AND “India”; “NREGA” AND “Odisha”/”Orissa” |

*Note: Orissa was replaced with other state names such as Chhattisgarh, Gujarat, Tamil Nadu.

**Supplemental Table 2: Stakeholders interviewed to understand the reasons for policy and programmatic changes in the states**

| Type of stakeholders | Chhattisgarh | Gujarat | Odisha | Tamil Nadu |
| --- | --- | --- | --- | --- |
| Government | 7 | 12 | 13 | 9 |
| Academia | 2 | 2 | - | 9 |
| Civil society and development partners | 8 | 3 | 4 | 6 |
| **Total** | 17 | 17 | 17 | 24 |

**Supplemental Table 3: Multiple determinants across sectors contributed to changes in height-for-age Z score between 2006 and 2016 in India**

|  | **Chhattisgarh** | **Gujarat** | **Odisha** | **Tamil Nadu** |
| --- | --- | --- | --- | --- |
| Maternal factors | 18.06 | 11.10 | 17.61 | 28.13 |
| Village factors | 12.20 | 3.04 | 30.84 | 11.26 |
| Household living conditions | 23.19 | 35.35 | 25.06 | 41.18 |
| Health and nutrition interventions | 27.73 | 1.03 | 10.90 | 19.44 |
| Unexplained | 18.82 | 49.49 | 15.59 | 0.00 |

| **Supplemental Box 1:** **Interview guide for key informants**  Introduction to the study: Thank you for taking time to speak with us. Before we start, we would like to brief you on the reason for this interview. In recent years, there has been an increase in attention and political commitment to reducing undernutrition globally. To understand what kinds of changes happened and what has led to those changes, we are conducting a series of studies on what are the stories of change in nutrition in India.  We developed a timeline to examine to what extent nutrition outcomes and indicators have changed in the last decade, and how nutrition sensitive programs have been implemented and have achieved coverage. The timeline for xx state shows that there has been progress in stunting reduction between 2006 and 2016; while some of the proximal issues that affect undernutrition (women with a healthy BMI, exclusive breastfeeding, care during delivery and postnatal care) have shown improvements, certain larger issues such as sanitation and women’s education show relatively less progress. However, all this knowledge is from what we gathered through documents, data, etc. that tell us what changed and by how much, but it does not tell us ***how.*** To understand how such changes came about, we want to interview people such as you who have the knowledge and experience of developments in the state.  Verbal Consent: This interview will take about 1 hour. We would like to take your permission to audio record this interview. The audio recording is solely for research purposes and will not be shared with anyone or be personally attributed to you. Do we have your consent to record the interview? Thank you.  Open-ended questions   - What do you think has changed in the state in the last 15 years since its inception in 2000? Do you think the lives of people have improved? How do you think that the lives of people were affected?  In what ways? [Probe for what it was like when they were there] - What do you think has changed in the nutrition landscape since 2000 (*Refer to the timeline*). - How has the engagement of different stakeholders such as government, civil society organizations, NGOs and Development Partners changed in the state over the years? And how has that worked for nutrition in the state? (*This might become a follow-up question depending on what is not answered to*). - What has been the government's role in enabling these changes that have taken place in the state? Can the changes be attributed to any specific actions taken by the government? |
| --- |

**Supplemental figure 2: Stunting decline between 2006 and 2016 in India**

Notes: AARR: Annual average rate of reduction; Telangana and Andhra Pradesh are not represented here as these two states were formed from Andhra Pradesh in 2014 and hence the stunting could not be compared between 2006 and 2016
